# Development and Sensing Properties Study of Underwater Assembled Water Depth-Inclination Sensors for a Multi-Component Mooring System, Using a Self-Contained Technique

**DOI:** 10.3390/s16111925

**Published:** 2016-11-16

**Authors:** Wenhua Wu, Jiaguo Feng, Bin Xie, Da Tang, Qianjin Yue, Ribin Xie

**Affiliations:** 1 State Key Laboratory of Structural Analysis for Industrial Equipment, Dalian University of Technology, Dalian 116024, China; yueqj@dlut.edu.cn; 2 China National Offshore Oil Corporation, Research Institute, Beijing 100028, China; fengjg@cnooc.com.cn (J.F.); xiebin@cnooc.com.cn (B.X.); 3 Department of Computer Science and Technology, Dalian University of Technology, Dalian 116024, China; tangda@dlut.edu.cn; 4 China National Offshore Oil Corporation, Shenzhen Branch, Shenzhen 518000, China; ribin_xie@cnooc.com.cn

**Keywords:** standalone, water depth-inclination sensor, multi-component mooring system, self-contained technique, offshore platform

## Abstract

Prototype monitoring techniques play an important role in the safety guarantee of mooring systems in marine engineering. In general, the complexities of harsh ocean environmental conditions bring difficulties to the traditional monitoring methods of application, implementation and maintenance. Large amounts of existing mooring systems still lack valid monitoring strategies. In this paper, an underwater monitoring method which may be used to achieve the mechanical responses of a multi-point catenary mooring system, is present. A novel self-contained assembled water depth-inclination (D-I) sensor is designed and manufactured. Several advanced technologies, such as standalone, low power consumption and synchronism, are considered to satisfy the long-term implementation requirements with low cost during the design process. The design scheme of the water resistance barrel and installation clamp, which satisfies the diver installation, are also provided in the paper. An on-site test has previously been carried out on a production semisubmersible platform in the South China Sea. The prototype data analyses, including the D-I value in the time domain (including the data recorded during the mooring retraction and release process) and spectral characteristics, are presented to reveal the accuracy, feasibility and stability of the sensor in terms of fitting for the prototype monitoring of catenary mooring systems, especially for in-service aging platforms.

## 1. Introduction

The safety of floating structures depends greatly on the accurate positioning of the mooring system under harsh environmental conditions. Multi-component mooring systems have been widely used as station keeping equipment for floating platforms in marine oil exploitation [1]. 

Model testing [2,3] is an essential component in the development and hydrodynamic verification of floating production systems. Recently, the above design methodologies have met difficulties involving the increasing water depth. The simplified model brings the uncertainties in the parameters and numerical modeling that are difficult to quantify into the calculation. Moreover, model tests have been challenged with the increasing water depth of marine resources. The traditional-scale model test cannot be conducted for deep water mooring systems. Stansberg et al. [4] claimed that there is no ocean laboratory which can be used to perform the testing of floating structures at depths greater than 1500 m for reasonable limits of model scale. This claim remains true today.

The prototype monitoring method for investigating the operation conditions of mooring line has been presented as an important method in the study of deep water mooring systems [5,6,7,8]. As the main straightforward measurement method used today, possessing low cost and good measuring accuracy, the top tension measurement of mooring lines by utilizing load cell sensors has been widely used in existing mooring systems [9,10,11]. However, the effects of the interaction between the mooring system and chain stopper leads to the decrease in the dynamic response of the mooring line gained by the load cell being smaller than the real value.

Van den Boom et al. [12] developed a monitoring system by using a preinstalled monitoring system to accurately acquire the top tension, moment and dynamic response of mooring systems. However, this type of measurement method is not suitable for existing platforms, due to the limitations of the installation method. The optical fiber technique was used by Smith and Williams [13] to establish the fatigue life of a synthetic fiber mooring line. The long-term reliability of the present methodology requires further verification in practical engineering applications.

In recent years, many additional measurement techniques and sensors for mooring systems have been developed. Du and Wu et al. [14] presented an underwater measurement method for investigating mooring line tension, based on the lumped mass finite element method. A standalone tilt sensor was designed and manufactured for recording the inclination varieties of the mooring line. The strong results of the restoring force and variations of the mooring line may be obtained using the present methods and sensors for the multi-point catenary mooring system, of which the length of the mooring line remains constant during the measurement process.

In the present paper, an underwater prototype monitoring strategy for multi-component mooring systems is presented. The governing equations for a three-component mooring system are presented in Section 2.1 showing the basic unknown variables and known conditions. The governing equations may easily be extended to other multi-segment mooring systems. In Section 2.2, by considering the requirements of the retraction and release of the mooring line during the well adjustment process, an underwater two-point prototype monitoring methodology is deduced. Two individual monitoring physical quantities (i.e., water depth and the inclination information in the article) were recorded together at each monitoring point to solve the other mechanical quantities related to the mooring lines. In order to meet the requirements of the underwater measurements of the water depth and inclination with high reliability, a self-contained sensor was designed and manufactured for recording the geometry varieties of multi-component mooring lines. The power module, storage module, water gauge module, tilt module and AD (analog digital) conversion module are integrated together in a water-protective sealed barrel. This conveniently protects the system from harsh ocean environment conditions, while also improving the sensor’s stability, reliability and durability. The proposed self-contained sensors have previously been implemented on a semi-submersible platform in the South China Sea. Valuable field test data from more than one month’s time have been recorded. The particular actions, such as the well-moving process or adjustments of the mooring line, can be traced using the recorded data. The spectral data analysis of the inclination and water depth are presented and discussed, to show the accuracy, feasibility and stability of the proposed sensor. The present integration methodology of the standalone sensor could be extended to other types of sensor designed with other concerned parameters in marine engineering [15].

## 2. Underwater Prototype Monitoring Strategy of the Multi-Component Mooring System

### 2.1. Governing Equations for the Multi-Component Mooring System

**Figure 1:**
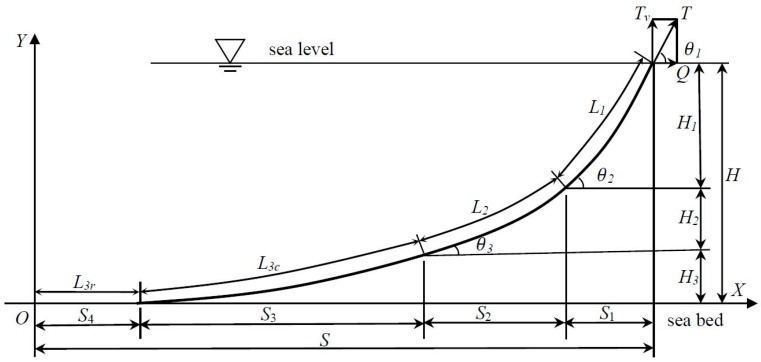
Layout of the three-component mooring system.

Without loss of generality, a three-component mooring system [1] is considered in this paper. The governing equations are easy to extend to other multi-component mooring systems. As shown in Figure 1, the mooring line should be located in the same plane, and elastic deformations are neglected during the geometry analysis.

The mooring line is connected with the floating platform through the fairlead, and expends in water to the seabed. The end touching point between the mooring line and seabed is marked as the coordinate origin (Point ‘O’ in Figure 1). The seabed is assumed to be planar, and marked as the *X*-axis. The *Y*-axis is perpendicular to the *X*-axis, and represents the water depth direction.

The governing equations of each component may be calculated via catenary equations, as follows:

(1) $$\tan\theta_{1}=\frac{w_{1}L_{1}+w_{2}L_{2}+w_{3}L_{3c}}{Q},\;\tan\theta_{2}=\frac{w_{2}L_{2}+w_{3}L_{3c}}{Q},\;\tan\theta_{3}=\frac{w_{3}L_{3c}}{Q},\;\tan\theta_{4}=0$$

(2) $$L_{3}=L_{3c}+L_{3r}\;T=\sqrt{Q^{2}+{\left(w_{1}L_{1}+w_{2}L_{2}+w_{3}L_{3c}\right)}^{2}}$$

(3) $$H_{k}=\frac{Q}{w_{k}}\left[\sqrt{\tan^{2}\theta_{k}+1}-\sqrt{\tan^{2}\theta_{k+1}+1}\right],\left(k=1,2,3\right)$$

(4) $$s_{k}=\frac{Q}{w_{k}}\left[\sinh^{-1}\left(\tan\theta_{k}\right)-\sinh^{-1}\left(\tan\theta_{k-1}\right)\right],\left(k=1,2,3\right)$$

where $L_{i}\left(i=1,2,3\right)$ are the lengths of the each components of the mooring line from top to bottom. $L_{3c},L_{3r}$ are the lengths of the lifting segment and residual segment on the seabed, respectively. $\theta_{i}\left(i=1,2,3\right)$ are the inclinations at the top of each component in respect to the *X*-axis, while $\theta_{4}$ is the inclination at the touch-down point, and is equal to zero. T is the tensile force along the mooring line, and its horizontal component (restoring force) is expressed as Q. $w_{i}\left(i=1,2,3\right)$ are the “wet” weights of each mooring component in water. Finally, $s_{i},h_{i}\left(i=1,2,3\right)$ are the lengths of each component mooring line in the horizontal and vertical (water depth) directions, respectively.

Observing the governing equations, namely Equations (1)–(4), it can be seen that there are a total of 12 equations with 20 unknown variables. Generally, the “wet” weight $w_{i}$ and length of each component $L_{i}$ are known variables, as the design indexes. Considering $\theta_{4}\equiv 0$, two additional variables must be measured to calculate the restoring force and other variables in Equations (1)–(4).

### 2.2. Underwater Prototype Monitoring Methodology

At present, there are three main types of monitoring methodologies used in multi-point mooring systems: (1) Load cells [12], which are installed at the chain stopper on the deck to measure the mooring line tension; (2) Fiber Bragg Gauges (FBG) [16], or strain gauges, which are pasted on the mooring line for short-term measurement; and (3) In-site underwater mooring systems [14], which have specially designed measurement instruments. 

Considering the requirements of the retraction and release of the mooring line during the well-moving process, the monitoring gauge cannot be allowed to be installed at the interaction point of the fairlead and first segment. Meanwhile, in order to facilitate the underwater operation of (Remote Operated Vehicles) ROVs or divers, especially to meet the needs of ease of the recovery and replacement, monitoring instruments must be installed at the first component of the mooring system. In this section of the paper, a novel underwater monitoring strategy, using a self-contained technique, is presented. 

**Figure 2:**
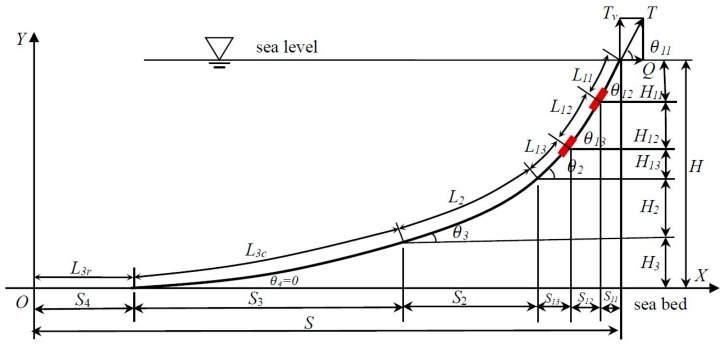
Underwater monitoring strategies with two self-contained sensors.

Two monitoring gauges are assumed to be installed at the first component of the mooring system (marked in red and shown in Figure 2). The data of the water Depth and Inclination (D-I) are acquired simultaneously at each installation location. In general, the lengths of $L_{11},L_{12},L_{13}$ could not be achieved with high precision. In this case, the entire mooring line could be regarded as a five-component system, with the following governing equations:

(5) $$\tan\theta_{11}=\frac{w_{1}\left(L_{11}+L_{12}+L_{13}\right)+w_{2}L_{2}+w_{3}L_{3c}}{Q}\;\tan\theta_{12}=\frac{w_{1}\left(L_{12}+L_{13}\right)+w_{2}L_{2}+w_{3}L_{3c}}{Q}\;\tan\theta_{13}=\frac{w_{1}\left(L_{13}\right)+w_{2}L_{2}+w_{3}L_{3c}}{Q}\;\tan\theta_{2}=\frac{w_{2}L_{2}+w_{3}L_{3c}}{Q}\;\tan\theta_{3}=\frac{w_{3}L_{3c}}{Q},\;\tan\theta_{4}=0$$

(6) $$L_{3}=L_{3c}+L_{3r},\;L_{1}=L_{11}+L_{12}+L_{13}$$

(7) $$T=\sqrt{Q^{2}+{\left(w_{1}\left(L_{11}+L_{12}+L_{13}\right)+w_{2}L_{2}+w_{3}L_{3c}\right)}^{2}}$$

(8) $$H_{11}=\frac{Q}{w_{1}}\left[\sqrt{\tan^{2}\theta_{11}+1}-\sqrt{\tan^{2}\theta_{12}+1}\right]\;H_{12}=\frac{Q}{w_{1}}\left[\sqrt{\tan^{2}\theta_{12}+1}-\sqrt{\tan^{2}\theta_{13}+1}\right]\;H_{13}=\frac{Q}{w_{1}}\left[\sqrt{\tan^{2}\theta_{13}+1}-\sqrt{\tan^{2}\theta_{2}+1}\right]\;H_{k}=\frac{Q}{w_{k}}\left[\sqrt{\tan^{2}\theta_{k}+1}-\sqrt{\tan^{2}\theta_{k+1}+1}\right],\left(k=2,3\right)$$

(9) $$s_{11}=\frac{Q}{w_{1}}\left[\sinh^{-1}\left(\tan\theta_{12}\right)-\sinh^{-1}\left(\tan\theta_{11}\right)\right]\;s_{12}=\frac{Q}{w_{1}}\left[\sinh^{-1}\left(\tan\theta_{13}\right)-\sinh^{-1}\left(\tan\theta_{12}\right)\right]\;s_{13}=\frac{Q}{w_{1}}\left[\sinh^{-1}\left(\tan\theta_{2}\right)-\sinh^{-1}\left(\tan\theta_{13}\right)\right]\;s_{k}=\frac{Q}{w_{k}}\left[\sinh^{-1}\left(\tan\theta_{k}\right)-\sinh^{-1}\left(\tan\theta_{k-1}\right)\right],\left(k=2,3\right)$$

As mentioned in Section 2.1, $w_{i}\left(i=1,2,3\right)$ and $L_{i}\left(i=1,2,3\right)$ are known variables. The governing equations (Equations (5)–(9)) include a total of 19 functions and 29 variables. Therefore, it may be observed that four monitoring physical quantities, which were obtained by two D-I gauges, provide sufficient conditions to satisfy the solving process of the governing equations (Equations (5)–(9)). Table 1 lists the summary of the all variables, including known variables, variables to be measured and variables to be solved.

## 3. Design of Underwater Standalone Water Depth-Inclination Sensor

In order meet the requirements of the underwater measurement of the water depth and inclination with high reliability, the self-contained technique was chosen and implemented in the design of the proposed sensor. The self-contained sensor has the abilities of independently collecting and recording geometry variations of the mooring line. It conveniently protects the system from harsh ocean environment conditions, while improving the sensor’s stability, reliability and durability. In order to satisfy the requirements of the on-site implementation, the following aspects should be considered in the design and manufacture of the sensor.

● Measurement capability of the sensors

The sensors must provide adequate measurement capability to obtain the complete measurement information time history. The parameters of the sensor, such as range, sampling frequency, frequency response and precision, must meet the measurement requirements with the global possible working conditions of the mooring line.

● Durability

Among the shortcomings of field monitoring are its unpredictability and uncontrollability of ocean environmental conditions. Long-term monitoring must be conducted to record large amounts of data by which to obtain the suitable data for analysis, including the data from harsh environmental states.

● Feasibility of field application

**Figure 3:**
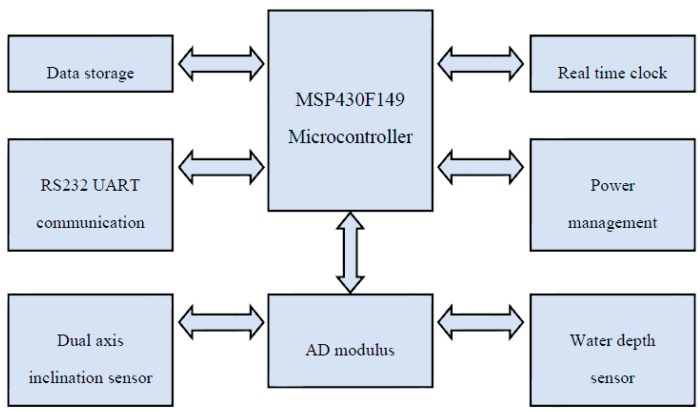
System block diagram of self-contained D-I sensors.

In order to collect the full range of mechanical response of the mooring system, the sampling frequency of the sensor, including water depth and inclination, must be adjusted from 1 to 10 Hz. Self-contained sensors are installed on the underwater mooring chains, and the requirements of the sensors being miniaturized and waterproof must be satisfied. The most suitable installation mode must also be considered. Figure 3 presents the system block diagram of the self-contained D-I sensors. The microprocessor, clock modules, water pressure and inclination modules, A/D transform, storage and power management are described in detail as follows:

(1) Microprocessor module

A MSP430F149 single-chip microcomputer with ultra-low power consumption is used throughout the device. The MSP430F149has 48 I/O (input/output) functional interfaces, among which eight I/O interfaces are used for external interrupt ports, while the others are available for simulating the INTER IC (IIC) and Serial Peripheral Interface (SPI) bus. This single-chip microcomputer is equipped with a piece of watchdog timer and two timer modules.

(2) Clock module

The time series is added to store information for data analysis in the future. A PCF8563 chip, produced by Philips Co. (Amsterdam, The Netherlands), is taken as the core chip of the clock module. The PCF8563 was powered by the wide voltage in the range of 1.0–5.5 V, with ultra-low power consumption, and the typical current was only 0.25 μA.

(3) Tilt module and A/D conversion module

For the tilt module, the MEMS tilt angle sensor chip produced by VTI Co. (Helsinki, Finland) is adopted. The range of the title sensor is from −90° to +90°. This chip can output two routes of tilt angle information of the *X*-axis and *Y*-axis in the form of a digital signal, and can also output the title information through the analog quantity in the form of 0.5–4.5 V voltage. Considering the fact that the response resolution of the analog signal can reach up to 0.0025°, which is better than that of the digital signal, two routes of analog voltage signals converted through A/D are adopted in this sensor.

(4) Water gauge module

**Figure 4:**
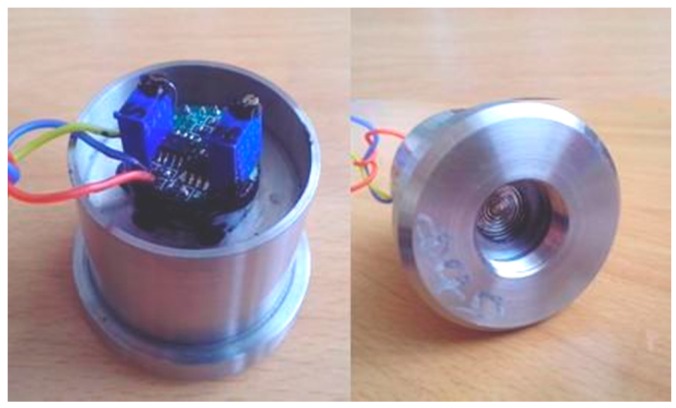
Water pressure modulus with sealing cover.

The CYY-6 water gauge is used as the probe of the water gauge, and is composed of a sensor and signal conditioning, as shown in Figure 4. It has a working voltage of 5 V, measuring range of 0–100 m, and compensation temperature range of −40–60 °C. The R&D Company (Lorton, VA, USA) was consigned to managing the overall pressure probe, conditioning module and subsequent calibration.

(5) Storage module

For the entire device, a Trans-Flash (TF) card with a high power capacity is taken as the storage card used for storing the information collected from the sensor. The information is stored in sections during storing.

(6) Power module

**Figure 5:**
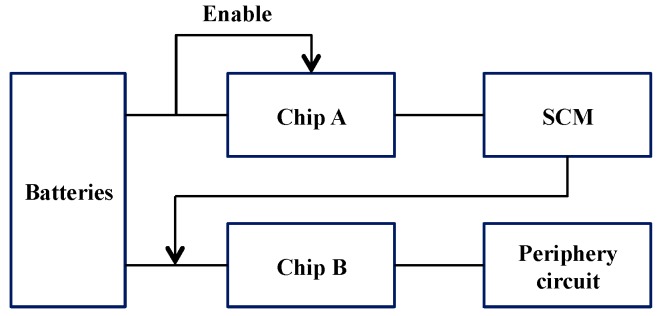
Schematic of the power management unit.

The entire self-contained underwater measuring device is powered by a lithium thionyl chloride battery. The single battery has a capacity of 19,000 mAh. It must be powered by two ER34615 batteries in series, due to the demands of the system’s working voltage. Figure 5 presents the schematic of the power management unit.

**Figure 6:**
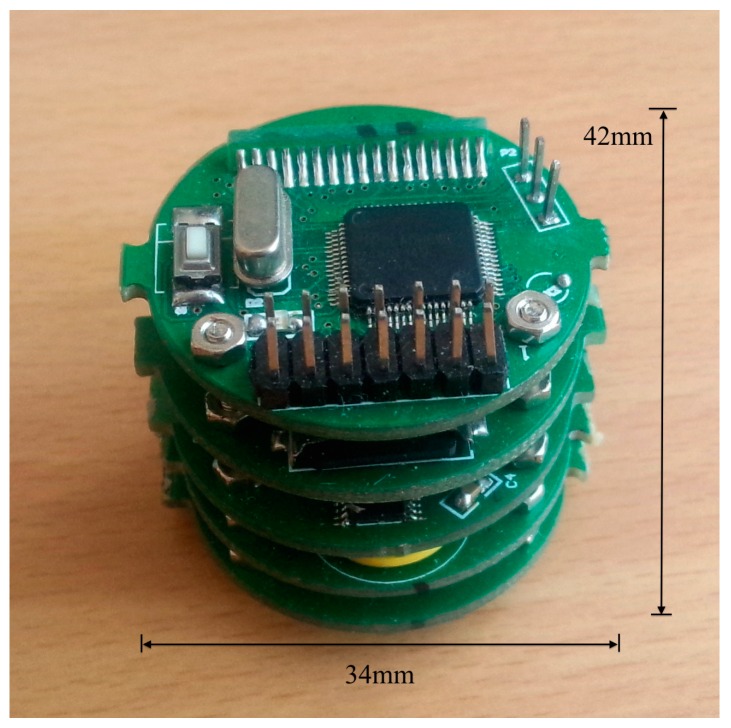
Integration of the circuits of the self-contained inclinometer.

The main design parameters of the self-contained sensor are listed in Table 2. The global integral circuits with the inclination modules are presented in Figure 6. The layouts of the circuits are consistent with a multi-layer structure. This allows convenience for the sensor assembling process, and saves much space. Furthermore, other functions may be easy to be established by the addition of a further layer.

## 4. Sensor Calibration, Field Test and Data Analysis

### 4.1. Protective Sealed Barrel and Installation Clamp

**Figure 7:**
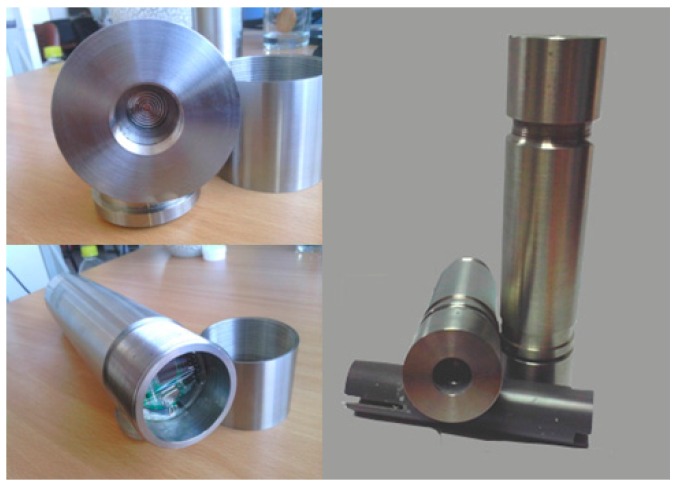
Self-contained sensors with water-resistant barrel.

A protective barrel was designed and manufactured to protect the sensor, so as to avoid the effects of water pressure and corrosion. The protective barrel was designed with a 320 mm height and 58 mm diameter, and can work at a water depth of 200 m. The final weight, as shown in Figure 7, is 2.6 kg, including the batteries.

**Figure 8:**
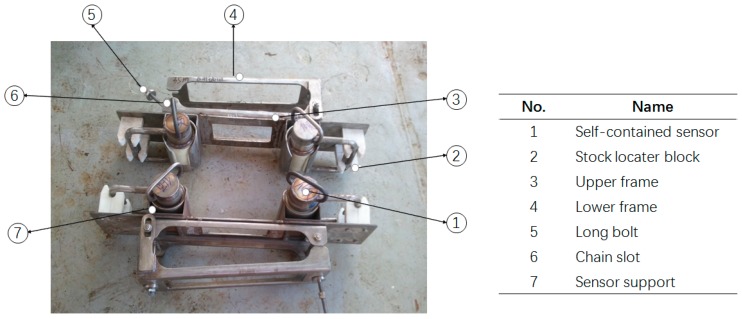
Specially designed clamp.

A special clamp was designed to fix the sensors on the mooring chains, as shown in Figure 8. The main components of the clamp are the upper frame, lower frame, long and chain bolts, stock locater block and sensor supports. The sensors may be installed by divers. The replacement and retrieval operations could be completed by ROVs and divers.

### 4.2. Sensor Calibration

**Figure 9:**
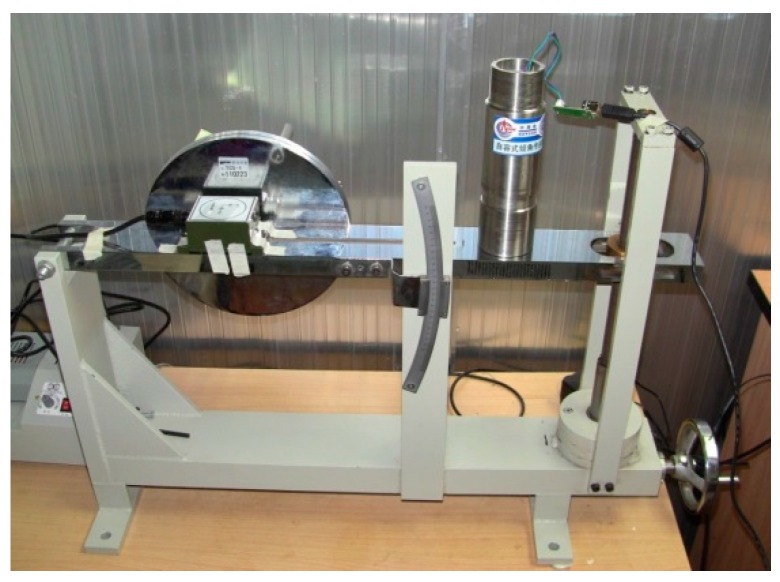
Inclination calibration system of the D-I sensor.

During the calibration of the D-I sensor, it is mainly the contents related to the angle of tilt that need to be measured, due to the fact that the pressure module of the sensor is ordered by the manufacturer. In general, three aspects, namely level zero drift, static measurement and dynamic measurement performances, must be considered in the period of using the inclinator. Therefore, the measurement of the self-contained tilt angle sensor is carried out specific to these three aspects. A Traction Control System (TCS) type tilt meter with a resolution of up to 9″ is adopted in the experiment system (as shown in Figure 9).

**Figure 10:**
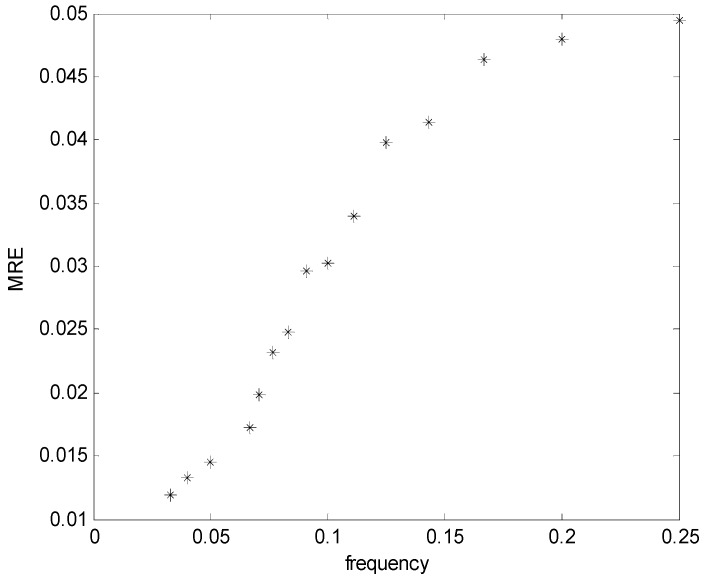
Dynamic response calibration of the D-I sensor.

(1) Level zero drift performance. A self-contained tilt angle sensor was placed on the platform at 0° for 24 h before the data were collected, with a collecting frequency of 1 Hz, then the fluctuating case of the data was observed. After many experiments, finally the measured drift distance of sensor was shown to be less than 0.01°, thus satisfying the measurement demands.
(2) Static measuring performance. The test board was used to set the different static angles, which were then measured with a self-contained tilt angle sensor. A group of typical measuring results is shown in Table 3. It is indicated that the maximum static angle difference of between self-contained tilt angle sensor and TCS tile meter is within 0.01°, thus meeting the design requirements.
(3) Dynamic performance test. The 6-Degree of Freedom (DOF) simulation platform was used to carry out the test experiment of the dynamic performance of the self-contained inclinator. The rotating amplitude of the platform was set as ±3°, and multiple frequencies from 0–0.25 Hz were set to conduct sinusoidal motion. The self-contained tilt angle sensor was used to measure the motion history to obtain the measurement amplitude, as shown in Figure 10.

From the test results, it can be seen that the results measured with the self-contained inclinator begin to generate deviation with the increase of the shaking frequency of the motion platform. This phenomenon is caused by the measurement principle of the inclinator. The posture of the mooring system mainly includes wave frequency and low frequency movement within 0.1 Hz, and the high frequency movement with about 0.1–0.15 Hz mainly includes the vibration with the small amplitude of the mooring system. Therefore, the dynamic testing results indicate that the system meets the accuracy requirements to measure the dynamic response of the mooring system with a self-contained inclinator.

### 4.3. Field Test and Data Analysis

**Figure 11:**
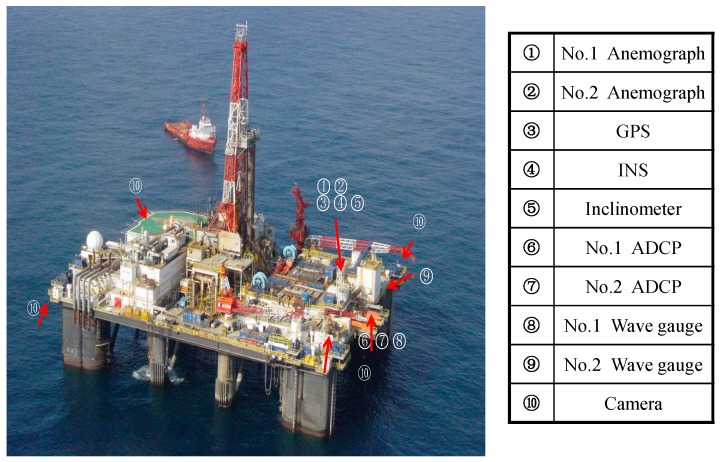
NHTZ FPS and sensors layout in JIP.

**Figure 12:**
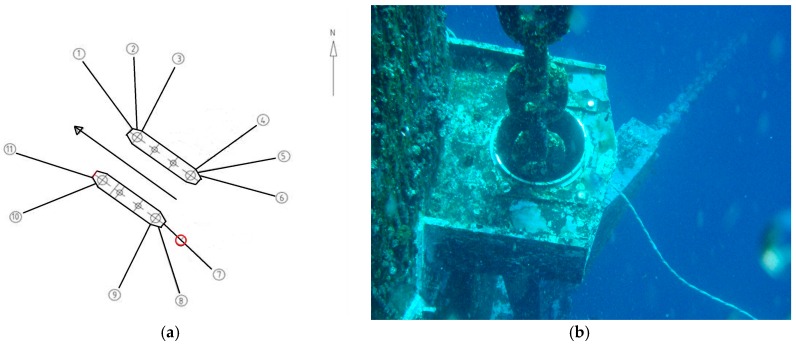
Mooring system configurations of NHTZ FPS (Installation position is marked with red circles). (**a**) Mooring line layout of NHTZ FPS; (**b**) Close-up view of the mooring lines.

The D-I self-contained sensors was tested and installed on the one floating production semisubmersible (NHTZ FPS) platform (Figure 11) in the South China Sea, sponsored by a prototype monitoring joint industrial project (JIP) [7]. A total of 11 asymmetric mobile spread mooring lines were installed in the FPS, as shown in Figure 12. Each mooring line was composed of three components. The corresponding parameters are shown in Table 4.

There are several points in the sensor installation process which require attention. First, the maximum installation water depth should be limited to within 50 m, due to the diver’s working ability; second, for the convenience of the well-moving and adjusting process, a certain amount of mooring line length should be retained according to the arrangements of the wellheads. The installation positions of two clamps should be arranged as far as possible from one another, to reduce the influence of the errors of inclination angle and water depth on the mooring restoring force calculation.

**Figure 13:**
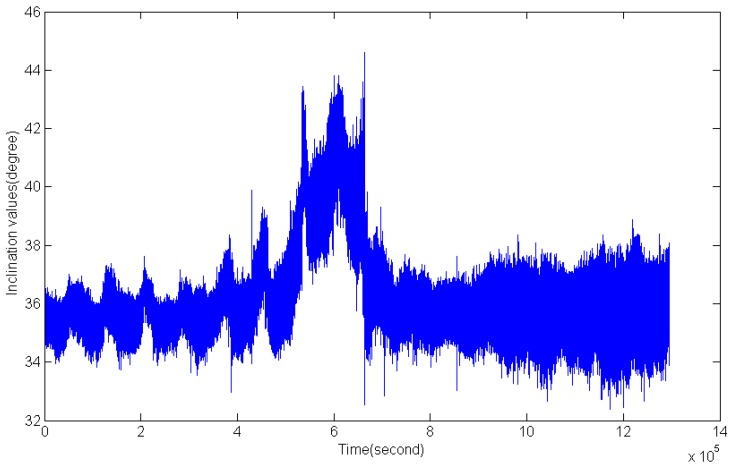
Inclination angle variations.

Due to the limitations of diving operation of the platform, only half a day was permitted to perform the field calibration of the sensor. Therefore, a D-I sensor was installed on the mooring line ⑦ from June to July 2015 (marked with red circles in Figure 13). The initial installation water depth was about 15 m.

**Figure 14:**
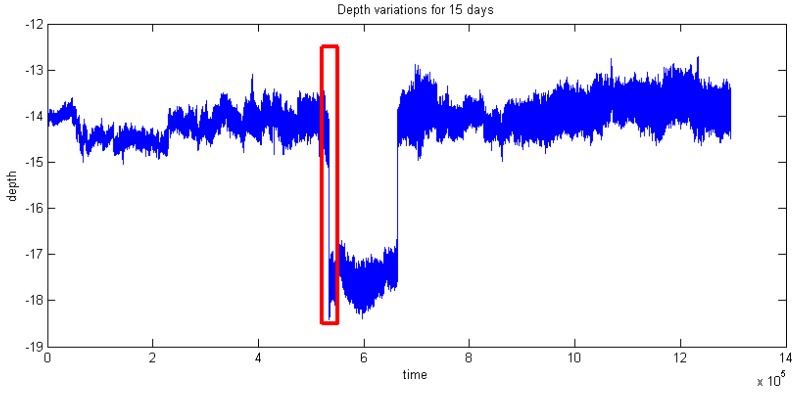
Water depth variations.

**Figure 15:**
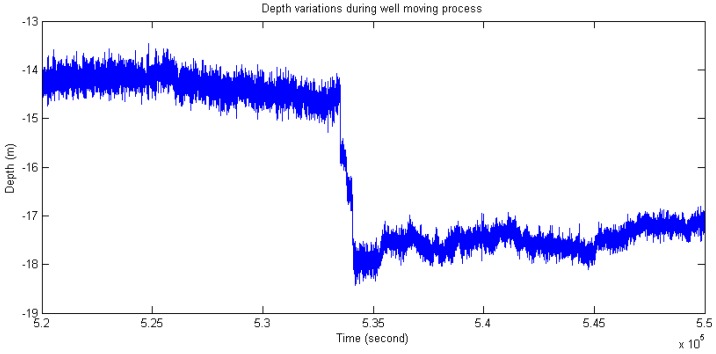
Variations of water depth during the mooring adjustment process (Close-up of red rectangle in Figure 14).

Figure 13 and Figure 14 illustrate the inclination angle and water depth distribution in the time domain within 14 days. In order to test the abilities of the present sensor in adapting to the changes of water depth and inclination, an adjustment process of the mooring line was carried out during the monitoring period. It could be observed that there were two sudden changes in Figure 13 and Figure 14. Figure 15 presents the partial enlarged image of the water depth profile at the rectangle range, marked with red in Figure 14.

**Figure 16:**
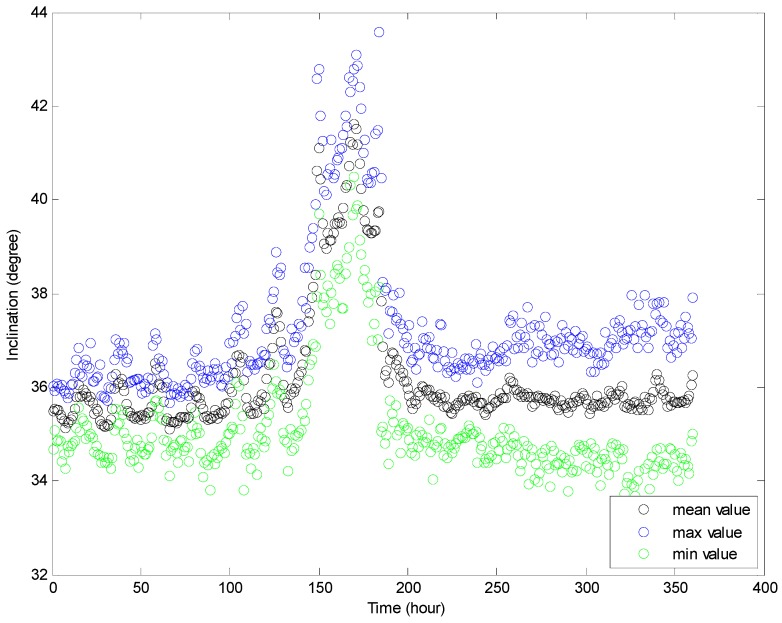
Maximum, mean and minimum values of inclination within each hour.

**Figure 17:**
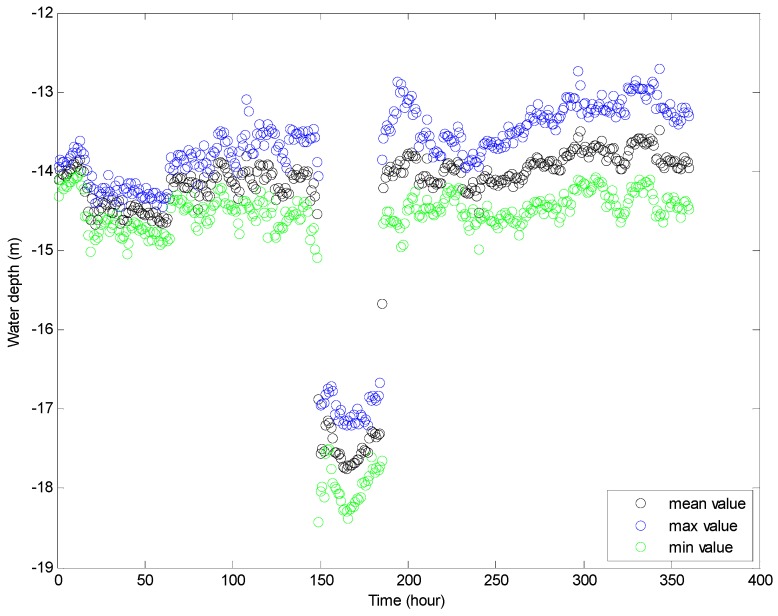
Maximum, mean and minimum values of water depth within each hour.

**Figure 18:**
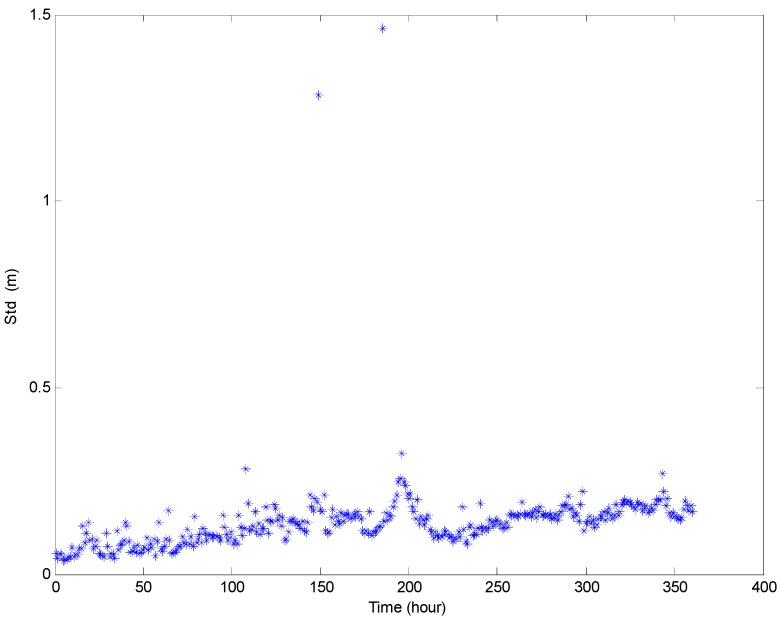
Standard deviation distributions of prototype monitoring water depth data per hour.

**Figure 19:**
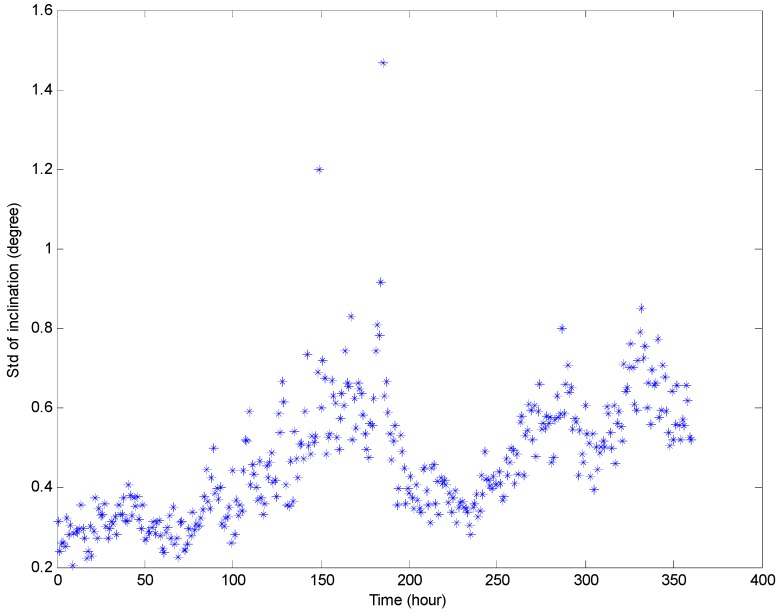
Standard deviation distributions of prototype monitoring inclination data per hour.

Figure 16 and Figure 17 show the maximum and minimum values of the water depth and inclination within each hour, respectively. The variation range of the inclination is about two to five degrees, and the variation range of the water depth within each hour is about 0.5–1.5 m in an ordinary operation condition state (except for the mooring adjustment process). Figure 18 and Figure 19 show the standard deviation distributions of the water depth and inclination within each hour. It could be observed that the standard deviation values of water depths are more consistent than those of the inclinations, except in the well moving process.

**Figure 20:**
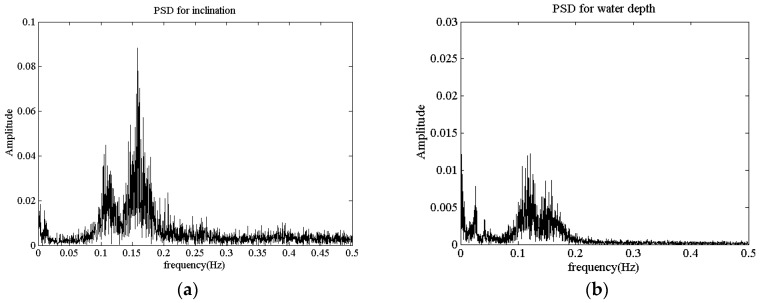
PSD results of inclination and water depth. (**a**) Inclination; (**b**) Water depth.

An important aspect of mooring system research is elucidating the contribution of the wave frequency components on the mooring line analysis. Figure 20a,b shows the distributions of the power spectral density (PSD) of thewater depth and inclination measured by the self-contained sensor. Two spectral peaks could be respectively found in the PSD figures at 0.12 Hz, which is close to the wave frequency, and at 0.16 Hz, which is near the natural frequency of the mooring line [16]. The PSD profiles of the present sensor show that the underwater field test system could accurately acquire the frequency domain characteristics of the mooring lines.

## 5. Conclusions

In this paper, a newly assembled underwater sensor was designed and developed to measure the mechanical response of a multi-component mooring line system in marine engineering. The water depth and inclination of the mooring line could be acquired synchronously. A self-contained technique was adopted in the sensor design and manufacture process. The structures of the integral circuits, configuration and parameters of the sensor were given in detail. A water-resistance barrel was designed with the water pressure detector. A special installation clamp was designed and manufactured to satisfy the requirements of installation by divers, and replacement and retrieval by ROVs. A prototype test was performed in NHTZ FPS in the South China Sea with 11 asymmetric three-component mooring systems. Nearly two months of water depth and inclination data were acquired from June to July 2015. Daily data analyses with mooring retraction and release process and spectral data analyses were provided to show the feasibility, accuracy and reliability of the present sensor in the measuring process of the mechanical response of the mooring line. The present integration methodology of the D-I sensor could be easily extended to similar types of self-contained sensors designed with other concerned parameters.

## Figures and Tables

**Table 1:** Summations of variables for governing equation.

Known Variables	Variables to Be Measured	Variables to Be Solved
$w_{i}\left(i=1,2,3\right)$	$\theta_{12},\theta_{13}$	$\theta_{11},\theta_{2},\theta_{3},$ T,Q, $H_{13},H_{2},H_{3},$ $L_{11},L_{12},L_{13},$ $L_{3c},L_{3r},$ $s_{11},s_{12},s_{13},s_{2},s_{3}$
$L_{i}\left(i=1,2,3\right)$	$H_{11},H_{12}$	
$\theta_{4}\equiv 0$		

**Table 2:** Major parameters of the self-contained sensor.

Parameter	Value
Inclination	−90°~90°
Water depth	0~100 m
Precision of water depth	0.1 m
Resolution of inclination	0.01°
Resolution of water depth	0.01 m
Designed life	>3 months

**Table 3:** Static inclination calibration.

**Angle Value**	**0°**	**0.05°**	**0.1°**	**0.5°**	**1°**	**3°**	**5°**	**7°**	**9°**
error	0.001°	0.004°	−0.003°	0.004°	0.007°	0.006°	0.009°	0.004°	0.008°
**Angle Value**	**0°**	**−0.05°**	**−0.1°**	**−0.5°**	**−1°**	**−3°**	**−5°**	**−7°**	**−9°**
error	0.001°	−0.002°	−0.002°	−0.005°	−0.006°	−0.009°	−0.005°	−0.009°	−0.010°

**Table 4:** Parameters of the three-component mooring line in the NHTZ FPS.

Mooring Component	Length (m)	Wet Weight (kg/m)
Mooring chain	220	274.61
Wire rope	450	70.72
Counterweight chain	600	369.09
